# Case Report of 49,XXXXY Syndrome: A Rare Variation of Klinefelter Syndrome With Seizure Disorder and ASD

**DOI:** 10.1002/ccr3.70257

**Published:** 2025-02-26

**Authors:** Ankit Shrestha, Biraj Parajuli, Aakash Pandit

**Affiliations:** ^1^ Department of Pediatrics Chitwan Medical College Bharatpur Nepal

**Keywords:** dural venous sinus aplasia, Fraccaro syndrome, paranasal sinus aplasia, X chromosomal aneuploidy, XXXXY syndrome

## Abstract

This case report presents a rare occurrence of 49,XXXXY syndrome in a 14‐month‐old male, the first documented case from Nepal, highlighting several distinctive clinical features. The patient had a height and weight below the third centile at birth and exhibited dysmorphic facial features, including a flat facial profile, flat nasal bridge, broad nose, low‐set ears, and clinodactyly, along with genital anomalies like micropenis and small testes. Neurologically, he demonstrated generalized hypotonia and global developmental delay. Atrial septal defect (ASD), left to right shunt, and mild tricuspid regurgitation were identified via echocardiography, adding to the complexity of the clinical presentation. Cytogenetic analysis of peripheral blood confirmed the 49,XXXXY karyotype in all 30 cells analyzed. The child also presented with a seizure episode at 11 months, a relatively uncommon manifestation in 49,XXXXY syndrome, which required symptomatic management. Neuroimaging revealed multiple abnormalities: A contrast‐enhanced computed tomography scan of the head showed mild hydrocephalus, while magnetic resonance imaging (MRI) findings included mild restricted diffusion in the bilateral frontal and parietal subcortical white matter, white matter volume loss around the lateral ventricles, and previously unreported anomalies, such as aplasia of the frontal and sphenoid paranasal sinuses and aplasia of the left transverse and sigmoid dural venous sinuses. These findings emphasize the need to recognize 49,XXXXY syndrome as a separate clinical entity from Klinefelter syndrome due to its unique features and severe cognitive and physical impairments. This case underscores the importance of comprehensive genetic evaluation and individualized, multidisciplinary management strategies for patients with rare chromosomal abnormalities. Further research is warranted to better understand the syndrome's unique clinical presentations and develop optimal therapeutic interventions.

**Summary**

- 49,XXXXY syndrome, while often grouped with KS, has distinct clinical and neuroimaging features that necessitate its recognition as a separate entity.
- Comprehensive genetic evaluation and tailored management are crucial in addressing the severe cognitive and physical impairments associated with this rare chromosomal anomaly.

## 1 Introduction

With a frequency of 1:500 males, Klinefelter syndrome (KS) is the most common sex chromosomal abnormality and is caused by the presence of one additional X chromosome (47,XXY) [1, 2].

Other X and Y polysomy, mosaicisms, and aberrant chromosomes have been reported, including 46,XX, 48,XXXY, 48,XXYY, 49,XXXXY, 47,XXY/48,XXXY, and 48,XXXY/49,XXXXY among others [3]. Although sharing some characteristics to KS and considered variants of KS, these do not correspond to KS, as they exhibit distinct phenotypes.

The 49,XXXXY syndrome was first described by Fraccaro et al., and it is the rarest and most severe variant phenotype of KS, with an incidence of 1:85,000 to 1:100,000 male births [4, 5].

It is characterized by a triad of hypogonadism, mental retardation, and musculoskeletal manifestations with some shared features to KS such as testicular dysgenesis and hypergonadotropic hypogonadism to KS [6].

Individuals with KS often face significant cognitive and behavioral challenges. These include language delays, such as difficulty with vocabulary, grammar, and complex language processing, along with executive dysfunction impacting attention, memory, and planning. Socially, they may exhibit withdrawn behavior and struggle with social cognition, including interpreting facial expressions and emotional cues. These difficulties contribute to academic struggles, low self‐esteem, and challenges in forming relationships. Psychologically, individuals with KS are at a higher risk for depression, anxiety, and psychotic disorders, necessitating tailored interventions from healthcare providers, educators, and families [7].

Individuals with intellectual disabilities (ID) face systemic challenges in criminal and civil law due to deficits in intellectual and adaptive functioning. Social biases and cognitive limitations can increase their vulnerability to exploitation and misunderstanding in legal processes. Their capacity for informed decision‐making in financial and medical contexts is frequently questioned, highlighting the need for targeted reforms, better societal awareness, and multidisciplinary support [8].

In this report, we present the case of a 14‐month‐old male child with a karyotype of 49,XXXXY, having some distinctive phenotypes and clinical presentations. The cytogenetic analysis of the peripheral blood revealed 49,XXXXY in 30/30 cells counted. To our knowledge, this is the first case reported from Nepal.

## 2 Case History/Examination

The patient was conceived spontaneously and born to a 23‐year‐old healthy mother in her first pregnancy via normal vaginal delivery at 37 weeks of gestation. At birth, his weight was 2.5 kg and his height was 45 cm, both of which were below the third percentile. There was no history of consanguinity among parents, and the pregnancy was uncomplicated, with no history of chromosome abnormality or consanguinity among family members. The father had an age of 27 years and presented no history of medical illness.

The postnatal period was uneventful. However, at 3 months of age, due to poor feeding and inadequate weight gain, the patient visited another tertiary care center where, on examination, it was reported a dysmorphic face including a roundish facial profile, 5th finger clinodactyly, flat nasal bridge, widening of the base of the nose, low‐set ears, short stature, micropenis, and small testes.

**FIGURE 1:**
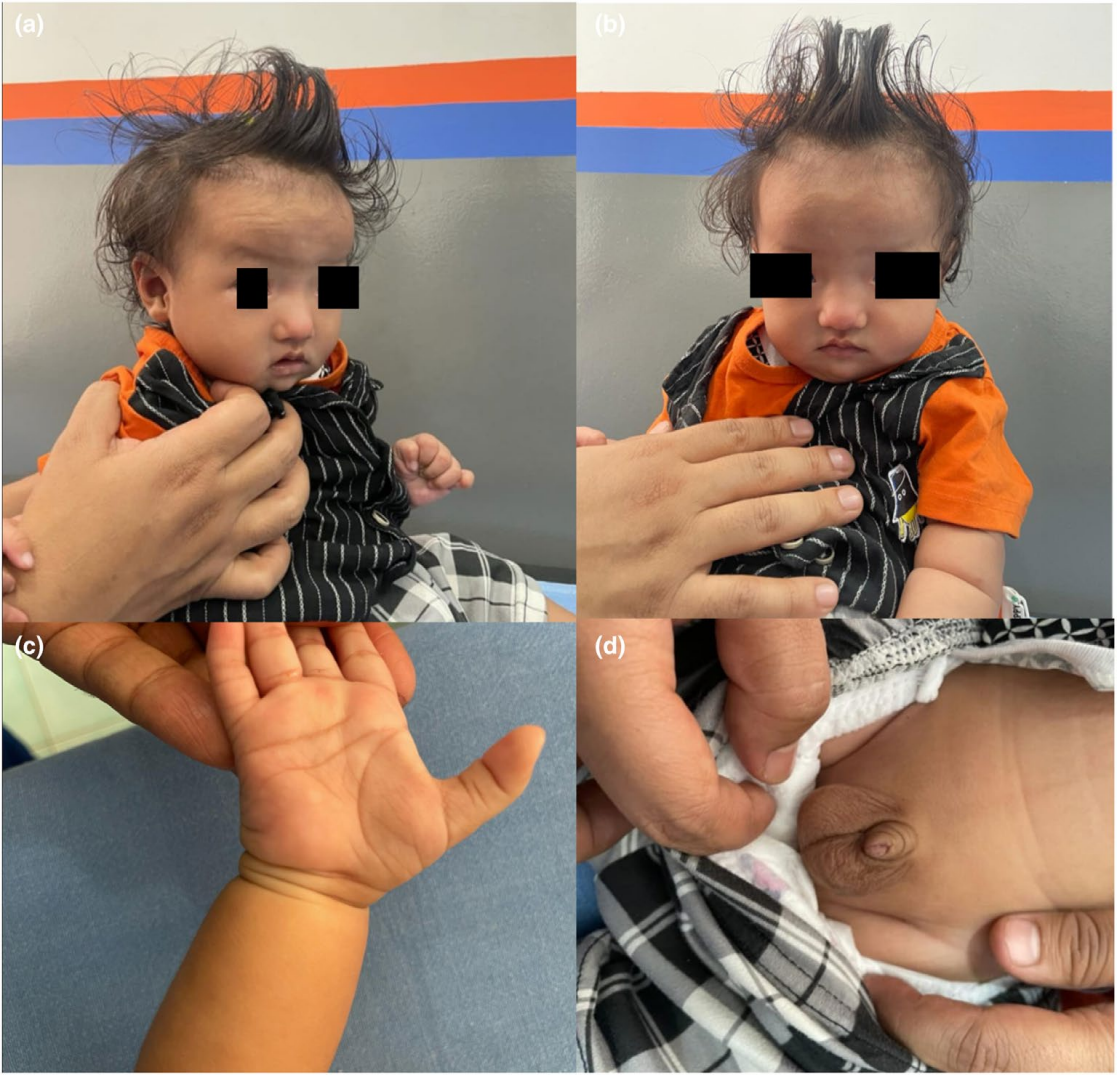
Clinical photographs. (a) Roundish facial profile. (b) Flat nasal bridge, Widening of the base of the nose, Low set ears. (c) 5th finger clinodactyly of the right hand. (d) Micropenis and small testis.

The stretched penile length was measured at 1.9 cm, which is below the reference range for the age group 1–3 years (2.4–3.5 cm) and close to the diagnostic threshold for micropenis (< 2.0 cm; approximately 2.5 standard deviations below the mean). Testicular volume was smaller than the smallest bead on the Prader orchidometer, < 1 mL, which is below the normative range of 1–2 mL for the age group (Figure 1). Neurologically, there was generalized hypotonia with no other gross musculoskeletal abnormalities. There is evidence of global developmental delay, with gross motor skills corresponding to a 9‐month level, and language, social‐adaptive, and fine motor skills corresponding to a 6‐month level.

## 3 Methods (Investigation and Treatment)

**FIGURE 2:**
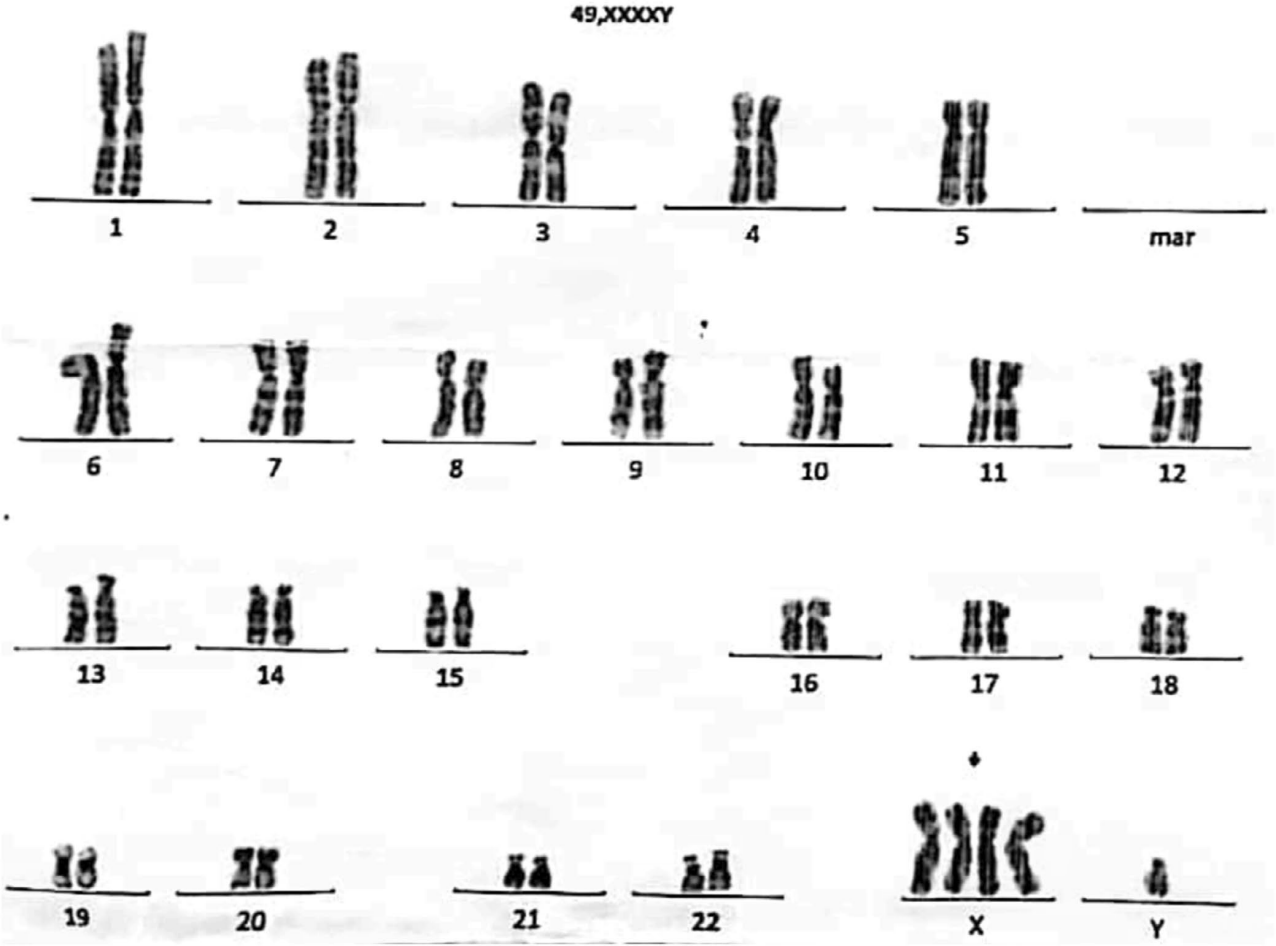
GTL‐Banding Karyotype showing an abnormal male karyotype with an additional three copies of chromosome X (49,XXXXY).

Echocardiogram was done with findings of secundum atrial septal defect (ASD) of 6.5 mm size (small (< 5 mm), moderate (5–10 mm), and large (> 10–20 mm)) with left to right shunt and mild tricuspid regurgitation (TR), tricuspid regurgitation peak gradient (TRPG) = 20 mmHg (normal range: ≤ 25 mmHg), left ventricular ejection fraction (LVEF) = 60% (normal range: 55%–70%). Analysis of metaphase chromosomes on peripheral blood cultures was performed. The cytogenetic analysis of the peripheral blood revealed 49,XXXXY in 30/30 cells counted (Figure 2). Several laboratory investigations on the sample of blood and CSF were done (Table 1).

**TABLE 1:** Laboratory investigations results.

Test name	Result	Unit	Reference range
Total leukocyte Count	4010	per mm^3^	4000–1000
Neutrophils	68	%	40–70
Lymphocytes	23	%	20–40
Eosinophils	0	%	2–6
Monocytes	9	%	2–10
Basophils	0	%	0–1
Platelets count	226,000	per mm^3^	150,000–350,000
Hemoglobin	12.1	g/dL	12–16
Red blood cell count	5	millions per mm^3^	0.5–5.5
Mean corpuscular hemoglobin	24.5	picograms	27–34
Mean corpuscular volume	78.3	Femtolitre	80–100
Mean corpuscular hemoglobin concentration (MCHC)	32	%	32–36
Serum sodium (Na^+^)	140	mmol/L	135–150
Serum potassium (K^+^)	4.8	mmol/L	3.5–5.5
Serum calcium (Ca^++^)	9.6	mmol/L	8–11
Serum magnesium (Mg^++^)	1.72	mmol/L	1.7–2.7
Serum creatinine	0.2	mg/dL	0.4–1.4
Blood urea	6.6	mg/dL	15–45
Blood sugar (random)	132	mg/dL	60–140
C‐reactive protein	2.0	mg/L	≤ 6 mg/L
CSF microprotein	23.0	mg/dL	15–45
CSF glucose	77.0	mg/dL	45–80
CSF WBC count	5	per mm^3^	0–5
CSF differential count			
Polymorph cells	0	%	Nil
Mononuclear cells	100	%	0–100
CSF culture & sensitivity	No organism isolated after 48 h of aerobic incubation at 37°C.		
CSF gram stain	Organism not seen.		
Free T3	2.06	pg/mL	1.2–4.1
Free T4	9.3	pg/mL	8.9–17.2
TSH	0.9	uIU/mL	0.3–4.5
Serum bilirubin (total)	0.3	mg/dL	0.4–1.0
Serum bilirubin (direct)	0.2	mg/dL	0.1–0.4
SGPT	21.5	IU/L	5–40
SGOT	34	IU/L	5–35
Serum alkaline phosphatase	103	U/L	30–120
Serum phosphorus	4.2	mg/dL	3.0–5.0

Global developmental delay (GDD) is defined as a substantial delay, measured as at least two standard deviations below the mean on standardized developmental assessments, in two or more developmental domains in children under 5 years old. However, this definition excludes children whose delays are primarily due to motor impairments or significant uncorrected visual or hearing deficits.

In our case, the child being aged 11 months exhibits features consistent with global developmental delay. Neurologically, there was generalized hypotonia without gross musculoskeletal abnormalities. Developmentally, gross motor skills were at the 9‐month level; for example, while an 11‐month‐old is typically able to stand momentarily without support and cruise along furniture, this child was only able to pull to stand and crawl. Fine motor, language, and social‐adaptive skills were more significantly delayed at the 6‐month level. For instance, an 11‐month‐old is expected to use a pincer grasp to pick up small objects and intentionally release them, yet this child relies on a raking grasp and struggles with object transfer. Similarly, in terms of language, an 11‐month‐old often says “mama” or “dada” meaningfully and responds to simple commands, but this child exhibits babbling without meaningful words or consistent responses to their name. Socially, while an 11‐month‐old typically waves “bye‐bye,” engages in games like peek‐a‐boo, and shows stranger anxiety, this child lacks these interactive gestures and exhibits less complex social interactions. These developmental discrepancies emphasize the extent of delay across multiple domains.

A fixed‐dose combination of diuretics furosemide and spironolactone (20 mg/50 mg) was initiated due to the progressive enlargement of the secundum atrial septal defect (ASD) from 4 mm at 3 months to 6 mm at 7 months and 6.5 mm at 11 months, as revealed from past medical records, indicating ongoing left to right shunting. While there was mild tricuspid regurgitation (TR) and normal pulmonary pressures (TRPG = 20 mmHg) at 11 months, the patient had a history of dilated right atrium (RA) and right ventricle (RV), and pulmonary hypertension at 3 months. The progressively enlarging ASD raised concerns about continued volume overload to the right heart, which could lead to right ventricular dysfunction or heart failure over time. As a prophylactic measure to manage potential fluid retention and to mitigate the hemodynamic burden on the right side of the heart, furosemide and spironolactone were started to prevent right heart failure and optimize cardiac function in the context of sustained right heart strain from the left to right shunt.

He was apparently well, but at 11 months of age, he again presented to the emergency department with chief complaints of acute onset of abnormal body movements, characterized by stiffening of the upper and lower limbs, uprolling of the eyes, clenching of the teeth and frothing from the mouth. The episode lasted for about 30 min. Injection diazepam 2 mg was administered intravenously stat. Later, a contrast‐enhanced computed tomography (CECT) of the head was performed, which showed mild hydrocephalus with an Evans index of 0.37 (marker of ventricular volume) and a widened anterior fontanelle. The Evans index is the ratio of the maximum width of the frontal horns of the lateral ventricles to the maximal internal diameter of the skull at the same level employed in axial CT and magnetic resonance imaging (MRI) images (0.20–0.25: normal; 0.25–0.30: possible or early ventriculomegaly; > 0.30: ventriculomegaly).

**FIGURE 3:**
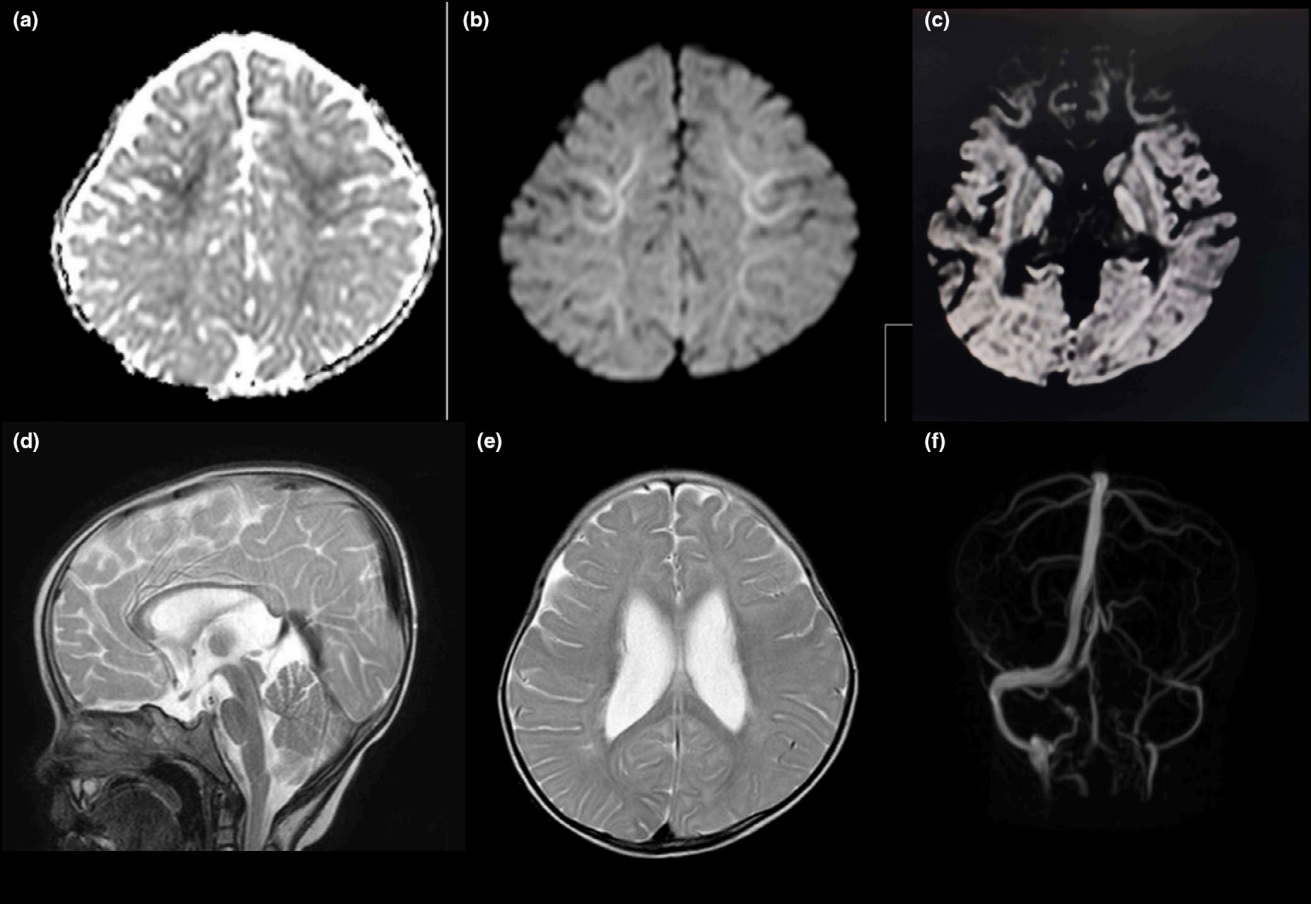
Magnetic resonance imaging (MRI) of the brain. (a) Apparent diffusion coefficient (ADC) showing mild restricted diffusion in subcortical white matter of Frontal Lobe (ADC is a measure of the magnitude of diffusion of water molecules within tissue, and is calculated using MRI with diffusion‐weighted imaging (DWI). (b) Diffusion‐weighted imaging showing mild restricted diffusion in subcortical white matter of Frontal Lobe. (c) Diffusion weighted imaging showing mild restricted diffusion in subcortical white matter of bilateral parietal lobe and globus pallidus. (d) T2 weighted MRI showing aplastic frontal (arrows) and sphenoid paranasal sinuses. (e) T2 weighted MRI showing mild white matter volume loss on the periventricular area of the lateral ventricle. T1 and T2 are basic pulse sequences on a MRI. A T1 MRI image supplies information about current disease activity by highlighting areas of active inflammation. A T2 MRI image provides information about disease burden or lesion load (the total amount of lesion area, both old and new). (f) Magnetic resonance (MR) angiogram showing aplasia of left transverse and sigmoid dural venous sinus.

**FIGURE 4:**
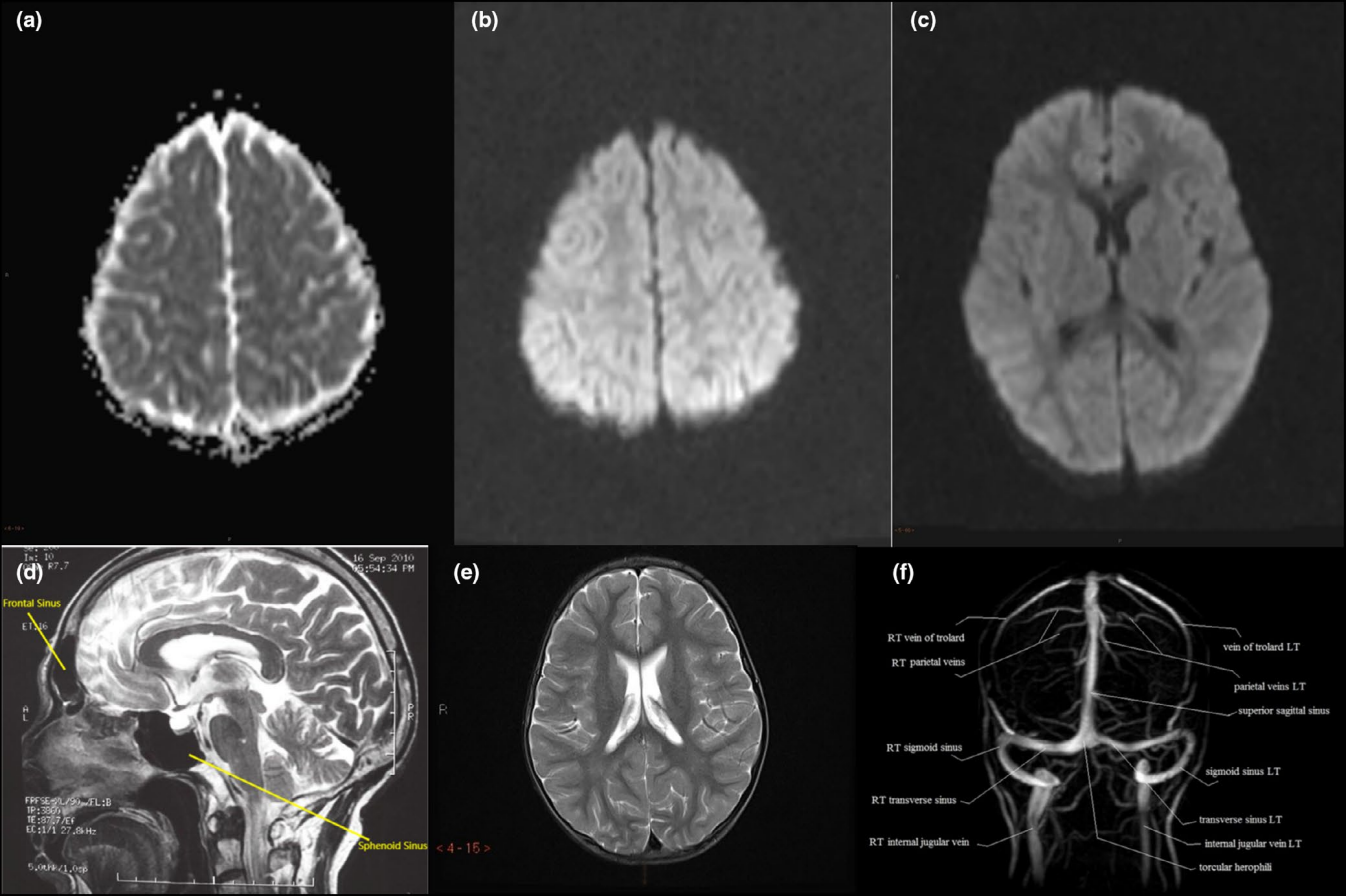
Control images of magnetic resonance imaging (MRI) of the brain. (a) Apparent diffusion coefficient (ADC) showing Frontal Lobe (ADC is a measure of the magnitude of diffusion of water molecules within tissue, and is calculated using MRI with diffusion‐weighted imaging (DWI). (b) Diffusion‐weighted imaging showing Frontal Lobe. (c) Diffusion weighted imaging showing bilateral parietal lobe and globus pallidus. (d) T2 weighted MRI showing frontal (arrows) and sphenoid paranasal sinuses (arrows). (e) T2 weighted MRI showing the periventricular area of the lateral ventricle. T1 and T2 are basic pulse sequences on a MRI. A T1 MRI image supplies information about current disease activity by highlighting areas of active inflammation. A T2 MRI image provides information about disease burden or lesion load (the total amount of lesion area, both old and new). (f) Magnetic resonance angiogram showing dural venous sinuses.

The MRI of the brain showed mild restricted diffusion in the bilateral frontal and parietal subcortical white matter and globus pallidus, suggestive of hypoxic damage with mild white matter volume loss in the periventricular area of the lateral ventricles. The apparent diffusion coefficient (ADC) is a measure of the magnitude of diffusion of water molecules within tissue and is calculated using MRI with diffusion‐weighted imaging (DWI). Diffusion‐weighted imaging is used for the detection of acute ischemia and to distinguish altered brain tissues. ADC values reflect the degree of diffusion of water molecules through different tissues (white matter: 670–800; deep gray matter: 700–850; cortical gray matter: 800–1000; 4 cerebrospinal fluid: 3000–3400). The MRI analysis also detected that the frontal and sphenoid paranasal sinuses were aplastic. Aplasia of the left transverse and sigmoid dural venous sinuses was also found on the magnetic resonance angiogram (MRA) (Figure 3). Control images of the respective sites were used for the analysis (Figure 4).

### 3.1 Conclusion and Results (Outcome and Follow‐Up)

The baby developed neuromuscular sequelae of the seizure with hypertonia, both axial (neck) and appendicular (lower limb), and developmental arrest without further seizure recurrence. Currently, the child is under occupational therapy for motor and speech therapy with improvement in the hypotonia.

## 4 Discussion

The 49,XXXXY chromosomal aneuploidy, also known as Fraccaro syndrome, is a rare condition often classified as a variant of KS. However, it should be diagnosed as an independent clinical syndrome as the two conditions have different etiologies as well as rather different clinical presentations [9].

In short, the 49,XXXXY syndrome is associated with below‐average height and frequent congenital malformations, while KS syndrome is characterized by above‐average height and rare congenital malformations. Mental and motor retardation are prevalent and severe in 49,XXXXY syndrome, with markedly low IQ (20–60), whereas only half of individuals with KS exhibit such impairments, with KS IQ typically ranging from 89 to 102. The 49,XXXXY syndrome is the rarest X chromosome aneuploidy, occurring in 1:85,000 males, while KS is the most common chromosomal anomaly in humans, affecting 1:500 men [1, 10, 11].

The KS and the 47,XYY syndrome are both common sex chromosome abnormalities in males, each occurring in 1:500 males. The KS is frequently diagnosed during infertility evaluation or gynecomastia, whereas the 47,XYY syndrome is often discovered incidentally, usually during genetic testing for behavioral problems. In addition to these syndromes, there are rarer sex chromosome numerical abnormalities in males, which include 48,XXYY, 48,XXXY, 49,XXXXY, and 49,XYYYY. A chromosome number (from the normal 16 chromosomes) exceeding 50 with six or more sex chromosomes has never been reported and is likely lethal [1, 11].

Each additional X chromosome results in a decrease in intellectual ability, with an approximate drop of 15–16 IQ points, particularly affecting language skills. Males with 48,XXYY tend to have higher IQs and better adaptation in daily living skills compared to those with 48,XXXY or 49,XXXXY, although all groups face significant challenges in communication and socialization. In conditions like KS and other X chromosomal aneuploidies, the presence of additional X chromosomes leads to decreased intellectual abilities, particularly in verbal and language skills, due to disruptions in gene expression caused by X‐chromosome inactivation (XCI) mechanisms and overexpression of genes that escape inactivation. Normally, XCI balances gene dosage, but in KS, genes such as GTPBP6 (guanosine triphosphate binding protein 6), EIF2S3 (eukaryotic translation initiation factor 2 subunit 3), and KDM5C (Lysine(K)‐specific demethylase 5C) escape inactivation and are overexpressed, contributing to cognitive deficits. GTPBP6 expression, for instance, negatively correlates with IQ, especially in individuals with random XCI patterns, while skewed XCI patterns result in milder cognitive deficits. Other genes like KDM5C and HUWE1 (HECT, UBA, and WWE Domain Containing E3 Ubiquitin Protein Ligase 1), involved in neuronal differentiation and synaptic plasticity, also contribute to impaired cognitive and language functions. About 15% of X‐linked genes escape inactivation, disrupting neural circuits for verbal and executive functions. Hormonal differences, particularly testosterone levels, further influence neurodevelopmental variability in KS [11, 12, 13].

Conversely, males with poly‐Y chromosomes, such as in the 49,XYYYY syndrome, do not exhibit as significant an intellectual deficiency as those with poly‐X chromosomes. Males with poly‐Y chromosomal arrangements, such as in the 49,XYYYY syndrome, typically exhibit milder intellectual impairment compared to those with poly‐X chromosomes. The additional Y chromosomes increase the expression of Y‐linked genes, such as SRY (sex‐determining region Y) and ZFY (Zinc finger protein, Y‐linked), which are involved in male differentiation and neurodevelopment. However, the smaller gene content and the absence of mechanisms like X‐chromosome inactivation (present in X polysomies) reduce the disruption to cognitive functions. Additionally, Y chromosome genes often have limited expression outside specific tissues, further minimizing systemic impact on intellectual abilities [11, 14].

Height is influenced by the number of sex chromosomes. The gain of an extra Y chromosome generally results in increased height, while the gain of an extra X chromosome tends to decrease height. The presence of three copies of the SHOX (short stature homeobox) gene, located in the pseudoautosomal region, contributes to increased height in individuals with KS. This is due to SHOX's role in promoting skeletal growth, particularly in the limbs. Excess SHOX expression explains the characteristic long leg length and arm span often exceeding height in KS patients. This genetic mechanism is independent of pubertal epiphyseal closure delays caused by relative hypogonadism. Polymorphisms in the length of CAG repeats in the androgen receptor (AR) gene influence phenotypic variability in KS. Longer CAG repeats are inversely correlated with AR function, affecting androgen‐mediated processes. This contributes to anthropometric variability, including correlations with increased height, arm span, and leg length, and delayed epiphyseal fusion. However, CAG repeat length shows inconsistent associations with other clinical features such as bone health, testicular volume, or cognitive outcomes [11, 13].

Fraccaro syndrome (49,XXXXY syndrome) has not been reported to be associated with maternal age or a hereditary disease [15].

Cognitive impairment along with language deficits, developmental delays, and speech dyspraxia is commonly associated with 49,XXXXY. Individuals with 49,XXXXY syndrome typically exhibit mild to moderate intellectual disabilities and face challenges in learning. Their speech and language abilities are significantly impacted, with a notable difference between their stronger ability to comprehend spoken language (receptive skills) and their difficulty producing speech (expressive skills). Many affected individuals are diagnosed with childhood apraxia of speech due to difficulties in coordinating the mouth movements required for speaking. Socially, these individuals often display shyness and a friendly demeanor. However, challenges in speech and communication can lead to behavioral issues, such as irritability, frustration intolerance, oppositional behavior, and temper outbursts. Physical symptoms of 49,XXXXY syndrome often include reduced muscle tone (hypotonia) and coordination issues, which delay developmental milestones like sitting, standing, and walking [16, 17]. In our case, the child had a global developmental delay.

Males with the 49,XXXXY syndrome present with distinctive facial features, such as round face, broad nasal bridge with epicanthic folds (abnormal narrowing of palpebral fissure; in many Asian populations, epicanthic folds are a normal anatomical variation due to differences in nasal bridge structure and periocular fat distribution. However, their presence in other populations, particularly with additional dysmorphic features, may suggest syndromes such as Down syndrome or other genetic conditions), micrognathia (mandibular hypoplasia is a condition in which the lower jaw is smaller than usual), hypertelorism (increased distance between the inner eye corners as well as the distance between the pupils), up‐slanting palpebral fissure (increased space between the eyelids), and low‐set ears with up‐slanting eyes (the ears are considered low set if the point at which the helix of the outer ear meets the cranium is at or below the line connecting the inner canthi of eyes). Skeletal system abnormalities include short stature, proximal radioulnar dysostosis (fusion of the proximal radius and ulna), vertebral anomalies (e.g., kyphoscoliosis), coxa valga (increased angle between the femoral neck and the femoral shaft), genu valgum (knee misalignment with the kneecap turned inward a condition in which the legs are bent inwards), and pes planus (flat feet, loss of the medial longitudinal arch of the foot) [18]. Most craniofacial abnormalities such as flat nasal bridge, widening of the base of the nose, and low‐set ears were identified in our patient, and the length of the patient was 69 cm, which was below the third percentile at birth with clinodactyly in the fifth finger.

Genital anomalies such as micropenis, hypospadias, small testes, hypogonadism, inadequate testosterone production, and infertility are common. In the 49,XXXXY syndrome, inadequate testosterone production is more prominent due to the increased number of X chromosomes, compared to other chromosome aneuploidy syndromes like 47,XXY, 48,XXYY, and 48,XXXY. In conditions like KS and other X chromosomal aneuploidies, the presence of additional X chromosomes leads to decreased testosterone production due to disruptions in gene expression caused by X‐chromosome inactivation (XCI) mechanisms and overexpression of genes that escape inactivation. The SRY gene, located on the Y chromosome, plays a critical role in normal testicular organogenesis, initiating male sexual differentiation. However, it is not the only factor involved in testicular development. Genes located on the X chromosome, such as the DAX1 gene, along with certain autosomal genes, have been identified to counteract testicular differentiation, influencing the formation and function of the testes such as testosterone production. This anti‐testes activity can lead to dose‐dependent disruptions in testicular development, causing gonadal dysgenesis. The DAX1 gene, located on the X chromosome, is particularly significant. While a single copy of DAX1 does not appear to have a major impact on either ovarian or testicular differentiation, the presence of a double dose of this gene (as seen in individuals with additional X chromosomes) exerts a potent anti‐testes effect. This results in severe testicular dysfunction, including seminiferous tubule dysgenesis, and contributes to infertility. For example, individuals with a 46,XY karyotype who gain an additional X chromosome, as in Klinefelter syndrome (47,XXY), often present with testicular underdevelopment and infertility. In more extreme cases, such as those with polysomy X (e.g., 48,XXXY or 49,XXXXY syndromes), the consequences are more severe, with affected individuals experiencing hypoplastic and underdeveloped genitalia, as well as infertility. For example, 49,XXXXY syndrome is frequently associated with small testes (observed in 94% of cases), small penis, hypoplastic scrotum (80%), and conditions such as cryptorchidism (30%). Some individuals also present with ambiguous genitalia, underscoring the profound effects of the extra X chromosomes and the associated overexpression of DAX1 [12, 19]. Micropenis with small testis was seen in our case.

Seizures have been reported at a rate of 10%–15% in the 49,XXXXY syndrome [10]. Our case later presented with episodes of generalized tonic–clonic seizures, which were managed symptomatically with an antiepileptic.

In the literature, MRI studies of KS and its variants revealed several consistent patterns. Classic KS (47,XXY) often shows smaller brain volumes and enlarged lateral ventricles. For more severe variants, such as 49,XXXXY, MRI findings frequently include significant white matter abnormalities and volume loss. Reports indicated that individuals with the 49,XXXXY syndrome may exhibit extensive white matter changes and considerable volume loss in various brain regions, particularly in the parietal lobes and cerebellum. MRI findings align with the severity of the chromosomal abnormality, emphasizing that the degree of X‐chromosome polysomy correlates with increased brain abnormalities [20, 21, 22]. In Klinefelter syndrome (KS) and other X‐chromosomal aneuploidies, structural brain abnormalities have been linked to dysregulated gene expression on the PAR1 region of the X chromosome. These regions, which escape X‐inactivation, contribute to altered neuroanatomy, including changes in brain volume, white matter structure, and ventricular enlargement. The presence of additional X chromosomes in these syndromes leads to disruptions in brain development, with more severe abnormalities seen in individuals with higher X chromosome polysomy [23].

Our case report is consistent with these descriptions. The patient demonstrated mild white matter volume loss and abnormalities indicative of hypoxic damage. Restricted water diffusion was observed in the bilateral frontal and parietal subcortical white matter, aligning with the reported MRI features of severe KS variants. These findings are in line with the literature, which associates white matter changes with increased X‐chromosome polysomy. However, our patient also presented with additional, less commonly described findings. These included mild hydrocephalus with an Evans index of 0.37, widened anterior fontanelle, aplastic frontal and sphenoid sinuses, and aplasia of the left transverse and sigmoid dural venous sinuses.

In the 49,XXXXY syndrome, congenital heart defects like atrial septal defect (ASD), ventricular septal defect (VSD), patent ductus arteriosus (PDA), Fallot's Tetralogy, and pulmonary stenosis have been reported [9]. The mechanism underlying congenital heart defects (CHDs) in 49,XXXXY syndrome (and other aneuploidy syndromes) remains poorly understood. While the development of the human heart is influenced by a complex array of signaling pathways and genetic factors, including those associated with chromosomal abnormalities, the specific genetic mechanisms in conditions like 49,XXXXY syndrome are not well defined. The high incidence of CHDs in aneuploidy syndromes suggests a potential role for gene dosage effects and chromosomal imbalances, but the multifactorial nature of these defects, compounded by the involvement of numerous genes across the genome, complicates efforts to pinpoint exact pathogenic mechanisms [24]. Among these, PDA is the most frequently reported congenital heart defect in 49,XXXXY syndrome, which was also present in the first case reported by Fraccaro; however, in our case, the child had ASD [4].

## 5 Conclusion

In conclusion, this case report sheds light on the rare occurrence of 49,XXXXY syndrome, a variant of Klinefelter syndrome, presenting with both seizure disorder and congenital heart disease (ASD). To the best of the author's knowledge, this is the first reported case from Nepal. The additional MRI findings contribute further complexity to the typical MRI abnormalities associated with KS syndrome variants and may suggest additional developmental anomalies or overlapping conditions not commonly detailed in the existing literature. It is necessary to understand the importance of comprehensive genetic evaluation and individualized management. Strategies for patients with complex chromosomal abnormalities, highlighting the need for interdisciplinary collaboration among healthcare professionals to address the needs of affected individuals. Further research and case studies are warranted to enhance our understanding of the unique clinical presentation and optimal therapeutic interventions for individuals with rare chromosomal variations like 49,XXXXY syndrome.

## Author Contributions

**Ankit Shrestha:** conceptualization, formal analysis, writing – original draft, writing – review and editing. **Biraj Parajuli:** conceptualization, supervision, validation, writing – original draft, writing – review and editing. **Aakash Pandit:** data curation, writing – original draft, writing – review and editing.

## Disclosure

All authors have declared that they have no financial relationships at present or within the previous 3 years with any organizations that might have an interest in the submitted work.

## Consent

Written informed consent was obtained from the patient's parents/legal guardian for publication and any accompanying images. A copy of the written consent is available for review by the Editor‐in‐Chief of this journal on request.

## Conflicts of Interest

The authors declare no conflicts of interest.

## Supporting information


Data S1

## Data Availability

The data that support the findings of this study are available from the corresponding author upon reasonable request.
