# Supplementary material for: Case Report of 49,XXXXY Syndrome: A Rare Variation of Klinefelter Syndrome With Seizure Disorder and ASD
Source: Clin Case Rep. 2025 Feb 26;13(3):e70257. doi: 10.1002/ccr3.70257 (PMC11865017; doi:10.1002/ccr3.70257)
Supplement: Supplementary file 1 — Data S1 [file CCR3-13-e70257-s001.docx]

**IMAGES**


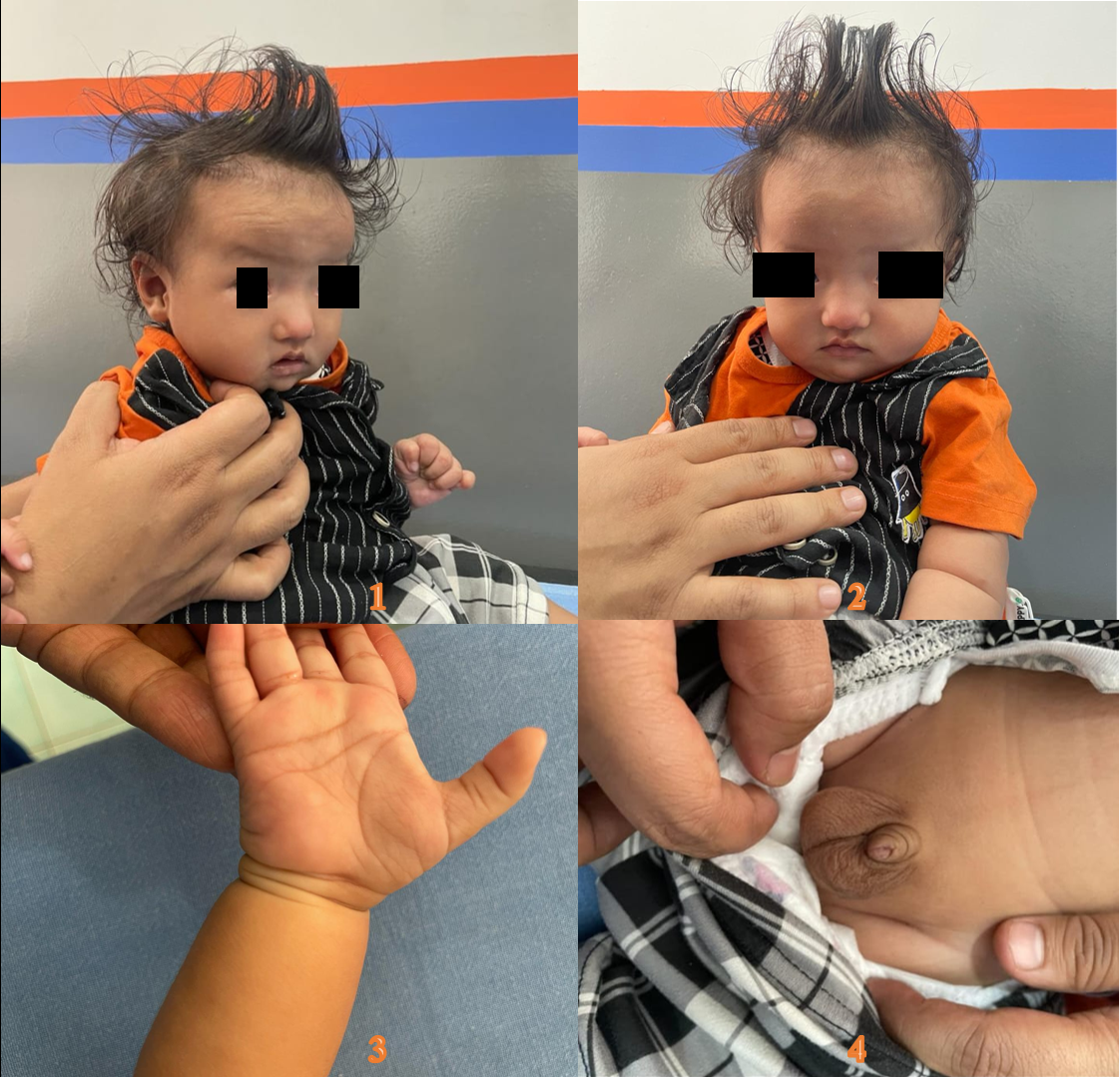


**Figure 1:** Clinical photographs. (1) Roundish facial profile (2) Flat nasal bridge, Widening of the base of the nose, Low set ears (2) Micropenis and small testis (3) 5th finger clinodactyly of right hand.


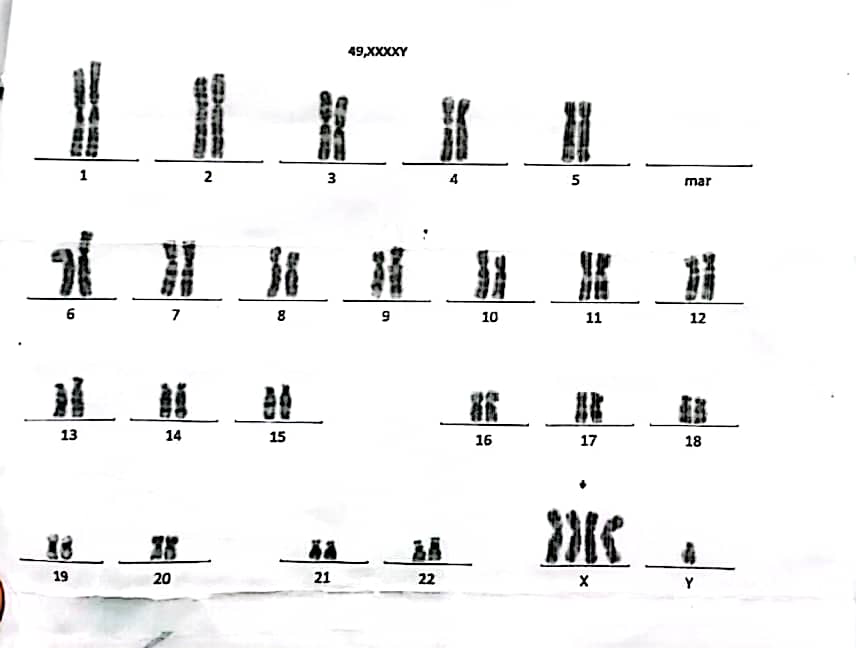


**Figure 2:** GTL-Banding Karyotype showing abnormal male karyotype with additional three copies of chromosome X (49,XXXXY).


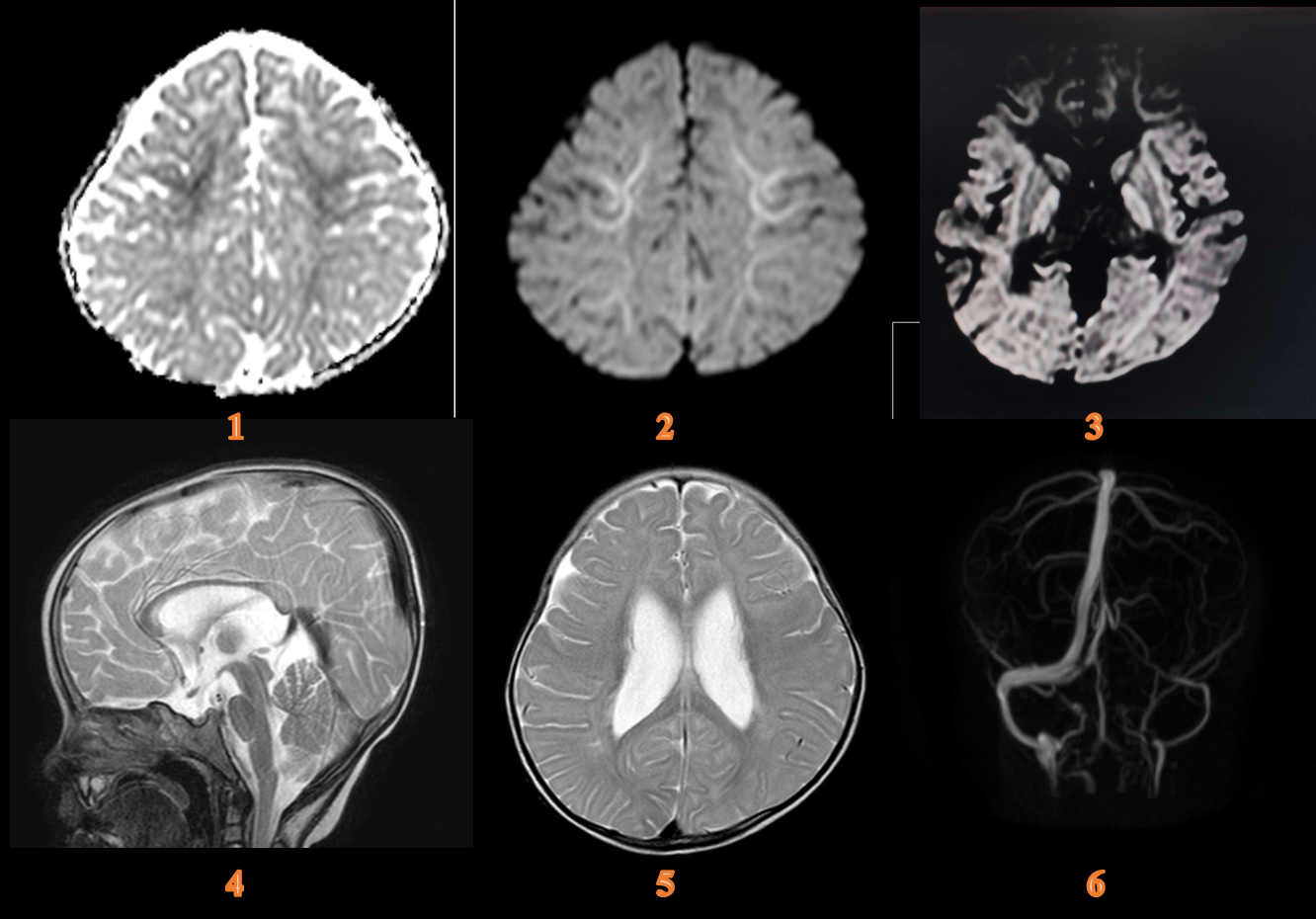


**Figure 3:** Magnetic resonance imaging (MRI) of the brain. (1) Apparent diffusion coefficient (ADC) showing mild restricted diffusion in subcortical white matter of Frontal Lobe (ADC is a measure of the magnitude of diffusion of water molecules within tissue, and is calculated using MRI with diffusion-weighted imaging (DWI). (2) Diffusion-weighted imaging showing mild restricted diffusion in subcortical white matter of Frontal Lobe. (3) Diffusion weighted imaging showing mild restricted diffusion in subcortical white matter of bilateral parietal lobe and globus pallidus. (4) T2 weighted MRI showing aplastic frontal (arrows) and sphenoid paranasal sinuses. (5) T2 weighted MRI showing mild white matter volume loss on the periventricular area of the lateral ventricle. T1 and T2 are basic pulse sequences on a MRI. A T1 MRI image supplies information about current disease activity by highlighting areas of active inflammation. A T2 MRI image provides information about disease burden or lesion load (the total amount of lesion area, both old and new). (6) Magnetic resonance (MR) angiogram showing aplasia of left transverse and sigmoid dural venous sinus.


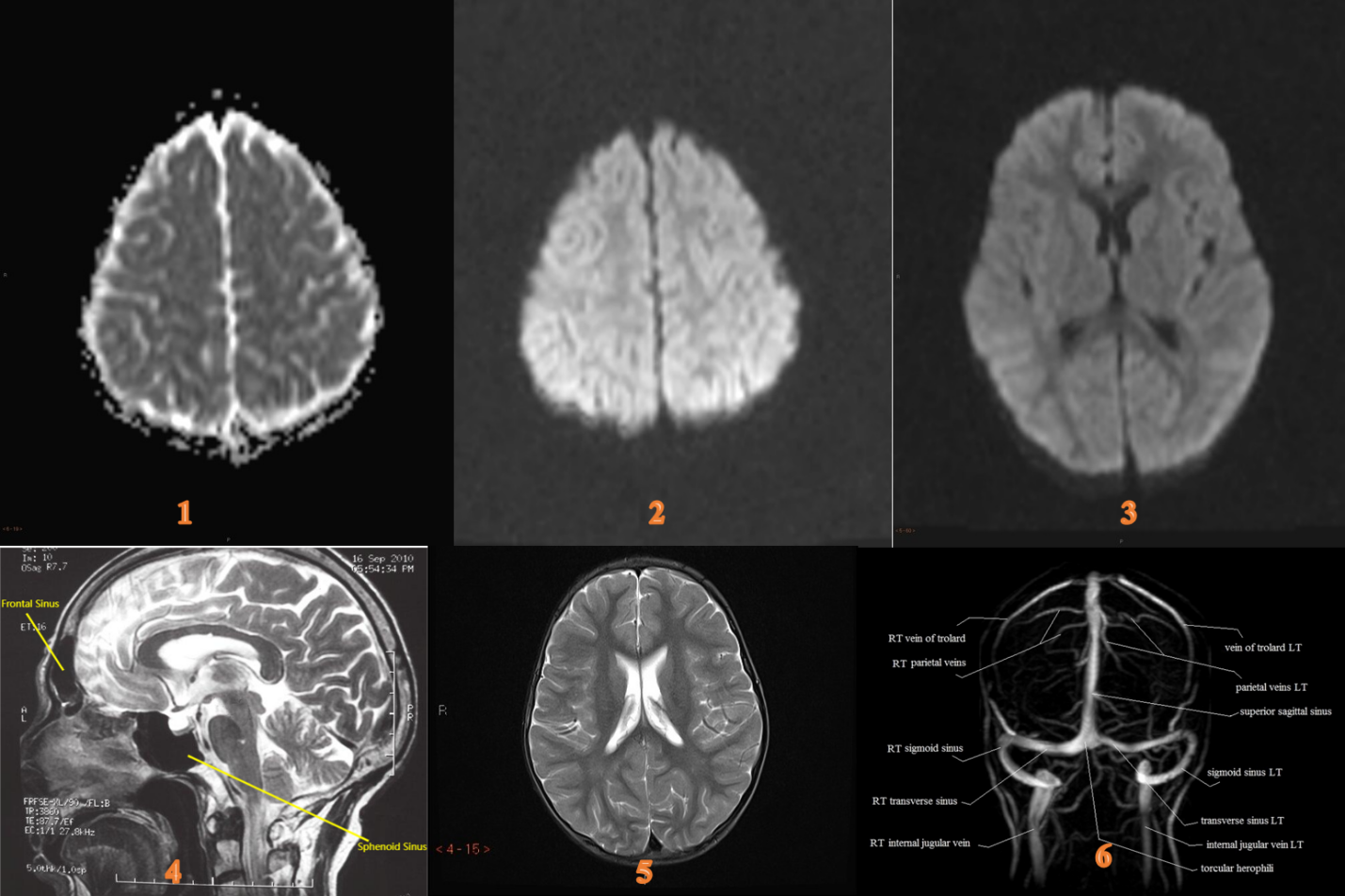


**Figure 4:** Control images of magnetic resonance imaging (MRI) of the brain. (1) Apparent diffusion coefficient (ADC) showing Frontal Lobe (ADC is a measure of the magnitude of diffusion of water molecules within tissue, and is calculated using MRI with diffusion-weighted imaging (DWI). (2) Diffusion-weighted imaging showing Frontal Lobe. (3) Diffusion weighted imaging showing bilateral parietal lobe and globus pallidus. (4) T2 weighted MRI showing frontal (arrows) and sphenoid paranasal sinuses (arrows). (5) T2 weighted MRI showing the periventricular area of the lateral ventricle. T1 and T2 are basic pulse sequences on a MRI. A T1 MRI image supplies information about current disease activity by highlighting areas of active inflammation. A T2 MRI image provides information about disease burden or lesion load (the total amount of lesion area, both old and new). (6) Magnetic resonance (MR) angiogram showing dural venous sinuses.

**TABLES**

| Test Name | Result | Unit | Reference Range |
| --- | --- | --- | --- |
| Total Leukocyte Count | 4010 | per mm^3^ | 4000-1000 |
| Neutrophils | 68 | % | 40-70 |
| Lymphocytes | 23 | % | 20-40 |
| Eosinophils | 0 | % | 2-6 |
| Monocytes | 9 | % | 2-10 |
| Basophils | 0 | % | 0-1 |
| Platelets Count | 226000 | per mm^3^ | 150000-350000 |
| Hemoglobin | 12.1 | g/dL | 12-16 |
| Red Blood Cell Count | 5 | millions per mm^3^ | .5-5.5 |
| Mean Corpuscular Hemoglobin | 24.5 | picograms | 27-34 |
| Mean Corupuscular Volume | 78.3 | femtolitre | 80-100 |
| Mean Corupuscular Hemoglobin Concentration (MCHC) | 32 | % | 32-36 |
| Serum Sodium (Na^+^) | 140 | mmol/L | 135-150 |
| Serum Potassum (K^+^) | 4.8 | mmol/L | 3.5-5.5 |
| Serum Calcium (Ca^++)^ | 9.6 | mmol/L | 8-11 |
| Serum Magnesium (Mg^++^) | 1.72 | mmol/L | 1.7-2.7 |
| Serum Creatinine | 0.2 | mg/dL | 0.4-1.4 |
| Blood Urea | 6.6 | mg/dL | 15-45 |
| Blood Sugar (Random) | 132 | mg/dL | 60-140 |
| C-Reactive Protein | 2.0 | mg/L | ≤6mg/L |
| CSF Microprotein | 23.0 | mg/dL | 15-45 |
| CSF Glucose | 77.0 | mg/dL | 45-80 |
| CSF WBC Count | 5 | per mm^3^ | 0-5 |
| CSF Differential Count | | | |
| Polymorph cells | 0 | % | Nil |
| Mononuclear cells | 100 | % | 0-100 |
| CSF Culture & Sensitivity | No organism isolated after 48 hours of aerobic incubation at 37°C. | | |
| CSF Gram Stain | Organism not seen. | | |
| Free T3 | 2.06 | pg/mL | 1.2-4.1 |
| Free T4 | 9.3 | pg/mL | 8.9-17.2 |
| TSH | 0.9 | uIU/mL | 0.3-4.5 |
| Serum Bilirubin (Total) | 0.3 | mg/dL | 0.4-1.0 |
| Serum Bilirubin (Direct) | 0.2 | mg/dL | 0.1-0.4 |
| SGPT | 21.5 | IU/L | 5-40 |
| SGOT | 34 | IU/L | 5-35 |
| Serum Alkaline Phosphatase | 103 | U/L | 30-120 |
| Serum Phosphorus | 4.2 | mg/dL | 3.0-5.0 |

**Table 1:** Laboratory investigations results
